# The value of long-term citizen science data for monitoring koala populations

**DOI:** 10.1038/s41598-019-46376-5

**Published:** 2019-07-11

**Authors:** Ravi Bandara Dissanayake, Mark Stevenson, Rachel Allavena, Joerg Henning

**Affiliations:** 1 School of Veterinary Science, The University of Queensland, Gatton, Queensland 4343 Australia; 2 Faculty of Veterinary and Agricultural Sciences, University of Melbourne, Parkville, Victoria 3010 Australia

**Keywords:** Ecological epidemiology, Ecological modelling

## Abstract

The active collection of wildlife sighting data by trained observers is expensive, restricted to small geographical areas and conducted infrequently. Reporting of wildlife sightings by members of the public provides an opportunity to collect wildlife data continuously over wider geographical areas, at lower cost. We used individual koala sightings reported by members of the public between 1997 and 2013 in South-East Queensland, Australia (*n* = 14,076 koala sightings) to describe spatial and temporal trends in koala presence, to estimate koala sighting density and to identify biases associated with sightings. Temporal trends in sightings mirrored the breeding season of koalas. Sightings were high in residential areas (63%), followed by agricultural (15%), and parkland (12%). The study area was divided into 57,780 one-square kilometer grid cells and grid cells with no sightings of koalas decreased over time (from 35% to 21%) indicative of a greater level of spatial overlap of koala home ranges and human activity areas over time. The density of reported koala sightings decreased as distance from primary and secondary roads increased, indicative of a higher search effort near roads. Our results show that koala sighting data can be used to refine koala distribution and population estimates derived from active surveying, on the condition that appropriate bias correction techniques are applied. Collecting koala absence and search effort information and conducting repeated searches for koalas in the same areas are useful approaches to improve the quality of sighting data in citizen science programs.

## Introduction

The koala (*Phascolarctos cinereus*), an arboreal folivorous marsupial, is an iconic Australian wildlife species^1^ living predominately in eucalyptus forests and woodlands of coastal areas of the states of Queensland, New South Wales (NSW), Victoria and South Australia. Koala populations are confined to unique habitats as their food resources are restricted to only 35 of the 600 eucalyptus species native to eastern Australia^2–4^. Movements of koalas are often affected by habitat fragmentation^5^ which compels them to move between trees on the ground, exposing them to attacks by predators like free-roaming dogs and to vehicular traffic, which often results in collisions and accidental deaths or injuries^6^. In addition, diseases such as chlamydiosis, which impairs the ability to breed, hotter and drier weather conditions and bush fires are threats to koalas^4,7–9^.

The koala is currently classified as ‘vulnerable’^10^ in South-East Queensland (SEQLD) as its distribution and abundance has been rapidly declining over the last few decades^11,12^. In 2010 the koala population in Queensland was estimated to be in the order of 167,000 which represents a 43% decline since 1990^12,13^. Due to this rapid decline koala conservation has gained wide attention from both state and federal governments and the general public. Koalas in the coastal areas of SEQLD have been protected with the implementation of the first koala conservation plan by Queensland Parks and Wildlife Services in 1995^14^.

Koala conservation efforts focus on reducing mortalities and addressing threats that impact on loss and degradation of habitat. For example, to prevent road deaths road signs have been erected along high risk areas to limit speed and to make motorists more vigilant about the presence of koalas crossing roads^15^. In addition, koala overpasses have been established in SEQLD to reduce the risk of vehicle collisions^16^. Further, government initiatives address koala habitat loss and habitat degradation through revised planning and policy frameworks^17^. This includes targeted efforts for the protection of existing koala habitat on public and private land and compensation for losses of koala habitat through habitat restoration^17^. Identification of the distribution of koala habitat and estimating koala abundance in different habitats are necessary to evaluate population trends relative to conservation efforts^18^. Such population monitoring is essential for evaluating the effectiveness of specific management and policy interventions and to assess progress towards achieving policy objectives^19,20^.

Estimation of koala populations in Australia is typically conducted by trained staff using ‘direct methods’ such as systematic field surveys in pre-identified areas (using either line or strip transect surveys or counting the total numbers of koalas in small survey areas) or ‘indirect’ methods such as observing scat counts under trees. Between 1996 and 2015 a total of 249 systematic surveys (each in an area of about 50 ha) were conducted to estimate koala population density in seven Local Government Areas (LGAs) of SEQLD^18^. While useful, systematic surveys are limited to small areas, not continued repeatedly throughout the year and are resource intensive to conduct. For these reasons, alternative approaches are required to continuously monitor koala populations.

Citizen science, the collection of data relating to observations made by members of the public, has the potential to provide a constant flow of data from larger geographical areas^21,22^. Citizen science is gaining momentum as a credible data collection technique due to the ubiquity of social media, smart phones and web technology, which provide economical and easily accessible tools for monitoring wildlife presence^23^. Examples of citizen science programs for collecting wildlife information include the North American Breeding Bird Survey (http://www.pwrc.usgs.gov/bbs/) which reports population changes and produces population maps based on sighting of more than 400 bird species, the global reporting of bird sightings on the eBird (http://www.ebird.org/) platform and the Frogwatch program conducted in the USA (http://www.aza.org/frogwatch). Some citizen data can guide further scientific research. Data obtained through the Frogwatch program, for example, are used by scientists to time surveys and to identify sampling sites to increase the probability of detection. While having the potential to provide a cost effective approach for documenting wildlife presence or absence appropriate adjustments need to be made to accumulated data to allow appropriate inferences to be made at the population level, in particular if chance observations are recorded without a specific sampling design^24–27^.

Often members of the public are keen to report dead, injured or animals under threat as they consider saving a wild animal’s life an act of humanity, especially for iconic native species such as the koala. This strong interest of the public in koala conservation can be utilized to promote data collection. For example, involvement of the public has been sought to monitor koalas by conducting scat surveys, by postal surveys or by reporting incidental sightings^28,29^. Well-planned and organized citizen science programs have the advantage that data can be collected using structured approaches. Such large-scale citizen science programs focusing on counting koalas have been carried out in South Australia in 2014 and 2016 and in NSW in 2013 and 2014^30^. The main objectives of these programs were to count the number of koalas in pre-identified areas using either mobile or web-based reporting tools.

Although no structured incidental koala count programs have been conducted in SEQLD, data on incidental koala sightings have been collected for more than two decades. In Queensland, incidental sightings, injuries, mortalities, and threats to koalas identified by members of the public are usually reported over the phone directly to the Department of Environment and Science (DES), local councils or to special interest groups working on koala conservation (such as wildlife carers) or the Royal Society for the Prevention of Cruelty to Animals (RSPCA). If a reported koala is ‘in danger’ rescue groups act promptly to rescue the koala and promptly return it to safe habitat. If a koala is sick or injured it will be submitted to veterinary facilities specializing in koala care, such as Moggill Koala Rehabilitation Centre located on the outskirts of Brisbane, the capital city of Queensland. Koala sightings, threats, rescue information, examination, treatment, and release details have been compiled in a database (KoalaBASE) developed by The University of Queensland’s School of Veterinary Science (www.koalabase.com.au) in collaboration with DES. Since 2014, only clinical records are compiled into KoalaBASE; details of koala sightings are entered in the WildNet database (http://www.data.qld.gov.au/dataset/qld-wildlife-data-api). Some councils in SEQLD have their own reporting mechanisms for koala sightings, either over the phone or using online forms. Some of these platforms are linked to the Atlas of Living Australia (www.ala.org.au) which aggregates biodiversity data from multiple sources and makes them freely available and usable online.

Identifying koalas in their natural habitat is challenging as they spend up to five hours during dusk and nighttime feeding on leaves^31,32^ and other times of the day and night sleeping and resting in trees^31,32^. Their lack of movement and grey fur provides excellent camouflage in eucalyptus tree canopies. The frequency of sightings can be influenced by habitat type, density, and location of the animal in a tree or on the ground, time of observation, and weather conditions. In addition, the frequency of sightings is influenced by the experience and interest of persons reporting a wildlife species^20,33^.

The aim of this study was to assess the quality and reliability of incidental koala sightings data collected in SEQLD over a period of two decades. We hypothesized that incidental sightings can be used to supplement and support structured koala population monitoring. Our analyses were conducted in two parts. Firstly, we described the spatial and temporal patterns of koala sighting densities using kernel smoothing. Secondly, we identified and quantified spatial biases associated with incidental koala sightings. For the latter, we considered koala sighting events as a spatial point process and quantified the association of koala sighting density with landscape features (such as distance to roads and koala habitat suitability).

## Results

### Overview of koala sightings

A total of 14,076 records on koalas sighted by members of the public in SEQLD were retrieved from KoalaBASE for the study period 1 January 1997 to 31 December 2013 (inclusive). For many koalas sighted, sex was not determined (57%, *n* = 7,866). Out of the sexed koalas, 50% (*n* = 3,076) were male and 50%, (*n* = 3,134) were female. Most of the koalas that were sighted were adults (83%, *n* = 11,708), followed by sub-adults (11%, *n* = 1,615) and young (1%, *n* = 104) while for some koalas, age information was not recorded (5%, *n* = 649).

**Figure 1:**
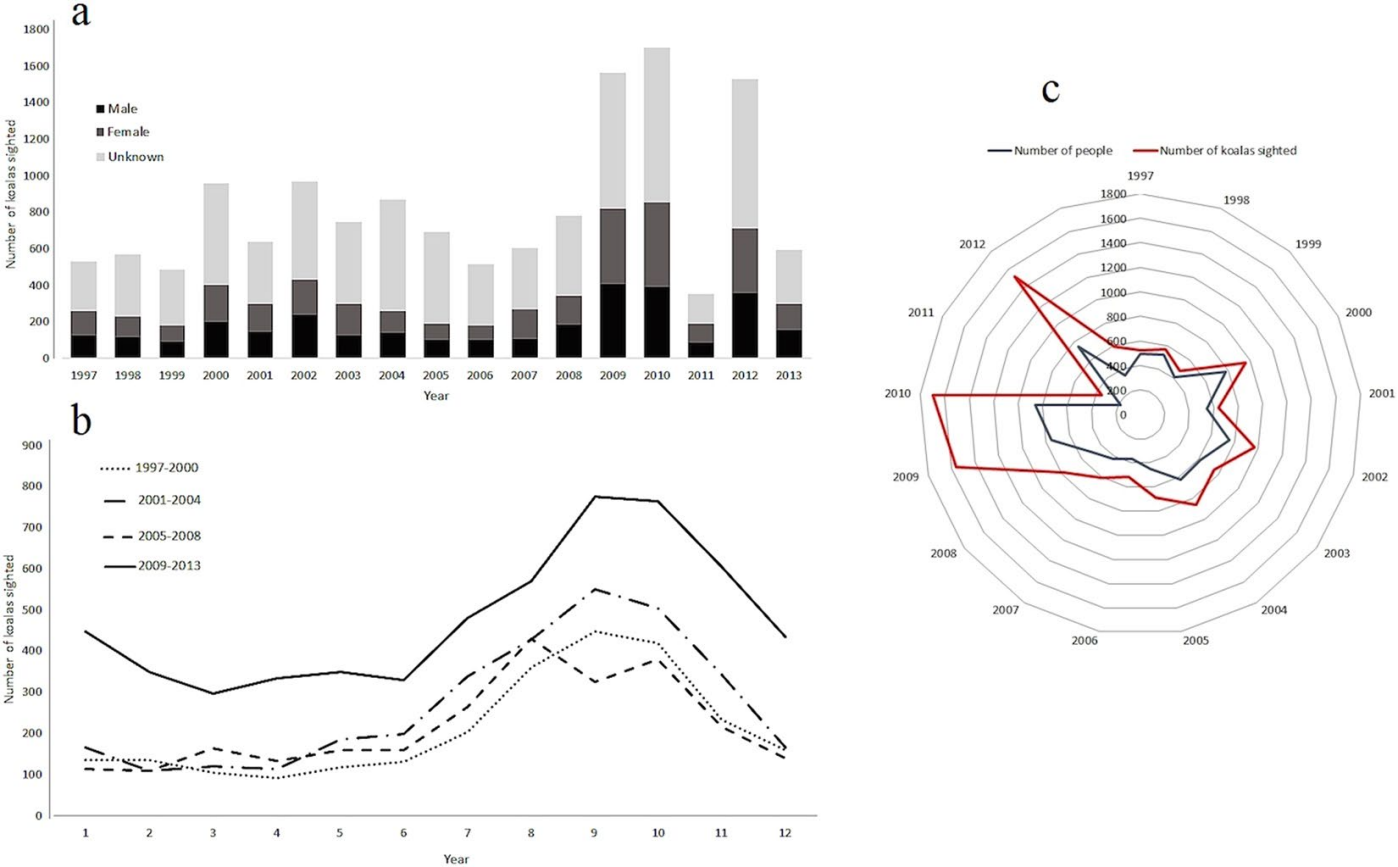
Temporal patterns of koala sightings between 1997 and 2013 in SEQLD, Australia. (**a**) Number of male, female koalas and of unknown sex sighted per year. (**b**) The number of koalas sighted in SEQLD as a function of calendar month for 1997–2000, 2001–2004, 2005–2008 and 2009–2013; and (**c**) Windrose chart showing the number of people and the number of koala sightings reported, 1997–2013.

Most sighting records (90%, *n* = 12,734) contained additional information related to where koalas were observed. The majority of koalas were seen in trees (87%, *n* = 11,090), while some were sighted on the ground (6%, *n* = 783) or in other locations such as on the rooves of houses, on fences, on roads or in garages (7%, *n* = 861). Each year, an average of 552 individuals (range 183 to 859) reported sightings with each member of the public reporting 1.4 (range 1.06 to 2.06) koalas per year. From 2009 to 2010 and in 2012 a small number of members of the public reported a large number (50 to 200) of sightings (Fig. 1c).

### Temporal patterns of koala sightings

The number of sightings per year fluctuated throughout the study period (Fig. 1a) with an average of 695 koalas sighted per year between 1997 and 2008. Major fluctuations included a rise in reporting in 2009–2010 (mean 1,630), low numbers being reported in 2011 (*n* = 353), an increase in reported sightings in 2012 (*n* = 1,527) and a further decrease in sightings in 2013 (*n* = 591).

There was a strong seasonal trend in koala sightings, with sightings increasing from June and peaking in September and October (Fig. 1b & Supplementary Fig. S3). Koalas were seen on the ground and in trees increasingly between July and November, coinciding with the breeding season.

**Figure 2:**
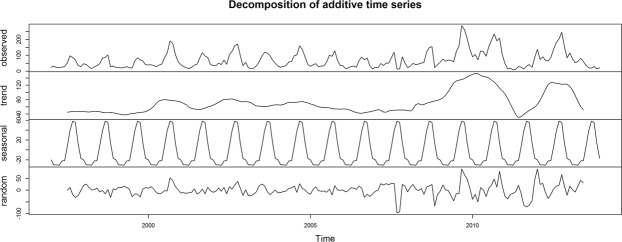
Decomposed time series of koala sighting data recorded between 1997 and 2013 in SEQLD, Australia. The panels from top to bottom show the original time series, the estimated trend component, the estimated seasonal component and the estimated irregular or random noise component. Trend components of the time series were prepared calculating simple moving averages in an order of 12 months.

Our time series analysis of the sighting data (Fig. 2) showed consistency in reporting of koala sightings between 1997 and 2008 and an increased trend from 2008 to 2010, low numbers in 2011 and then a return to a similar level in 2012 as in the 2008–2010 period. Random noise of koala sightings was, in general, relatively constant until 2008, while fluctuating markedly after 2008. The time series analysis highlighted a strong seasonal variation in koala sightings each year.

### Spatial patterns of koala sightings

**Figure 3:**
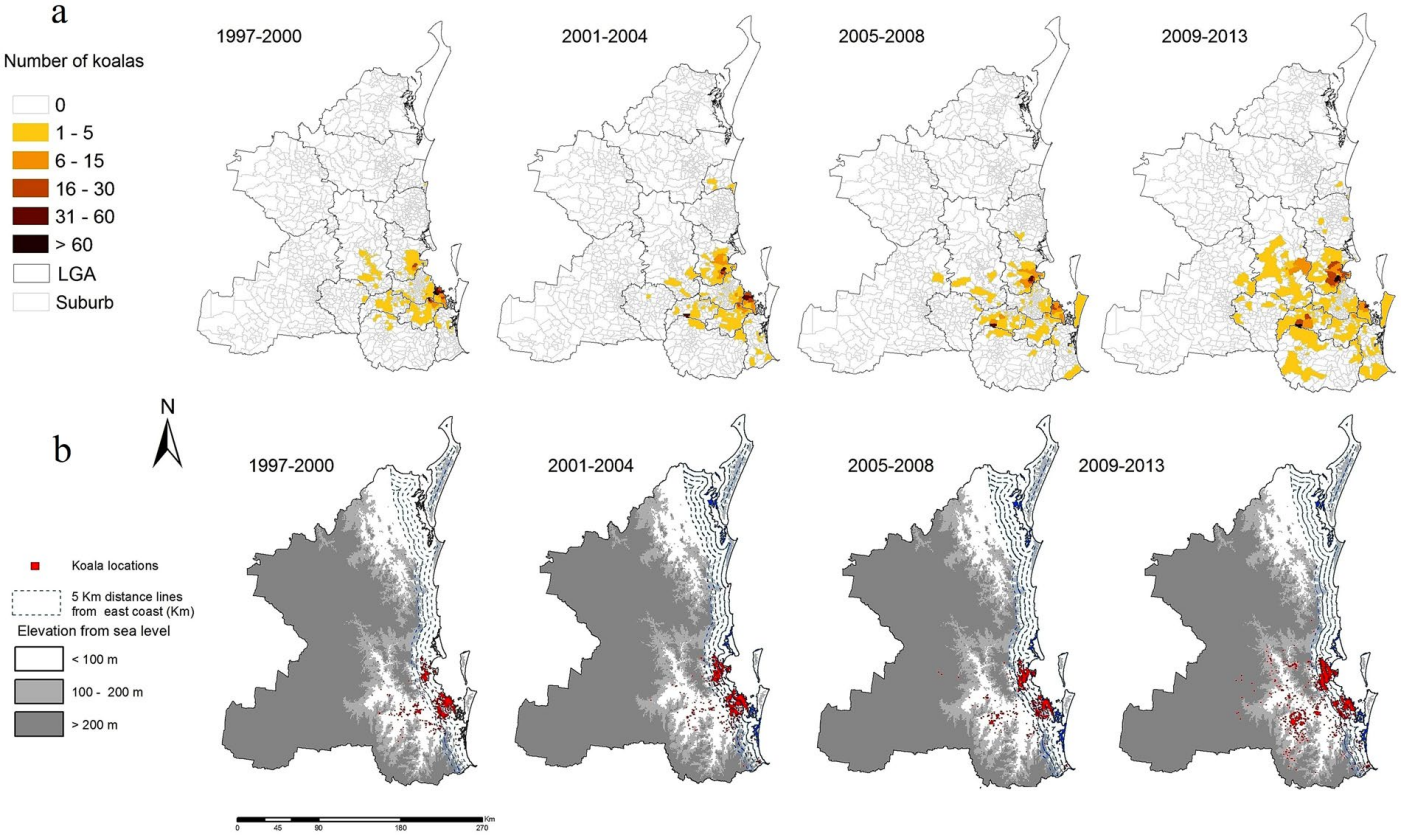
Spatial patterns of koala sightings between 1997 and 2013 in SEQLD, Australia. (**a**) Choropleth maps showing the number of koala sightings at LGA and suburb level, 1997–2000, 2001–2004, 2005–2008 and 2009–2013. (**b**) Point maps showing the location of individual koala sightings in relation to elevation (in meters) and distance to coastline. The data used to produce these maps were from KoalaBASE (https://www.koalabase.com.au), and Australia GIS map data are from Queensland spatial catalogue (http://qldspatial.information.qld.gov.au). Maps were generated using ESRI ArcGIS Desktop v10.5.0^47^ (ESRI Redlands, California, www.esri.com).

The majority of koala sightings (97%) in SEQLD were reported from five LGAs: Moreton Bay (44%), Redland (24%), Ipswich (12%), Logan (11%), and Brisbane (6%) (Fig. 3a and Supplementary Tables S7 & S8). The decrease in reported sightings in 2011 (Fig. 1a) was due to fewer reported sightings from Moreton Bay and Redland (a reduction in the frequency of 2011 sightings by 88% in Moreton Bay and 97% in Redland compared with 2010).

**Figure 4:**
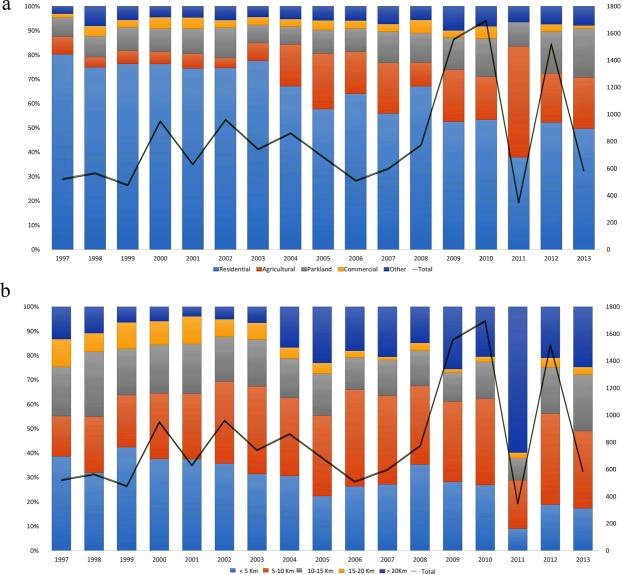
Frequency of koala sightings per year by land type between 1997 and 2013 in SEQLD, Australia. (**a**) Stacked bar chart showing the proportion of sightings occurring in different land types as a function of year. (**b**) Stacked bar chart showing the proportion of sightings occurring at categories of distance from the coast as a function of year.

Koala sightings were not reported from the far north and far western parts of SEQLD (Fig. 3). Locations of koala sightings expanded in the western and south western direction between 1997 and 2013 (Fig. 3a). Interestingly, 83% of sightings were reported within 20 km of the coastline (Figs 3b and 4b): approximately 29% of sightings (*n* = 4,090) were observed <5 km, 32% (*n* = 4,463) within 5–10 km, 17% (*n* = 2,421) within 10–15 km, 5% (*n* = 698) within 15–20 km distance from coastline. Most koala sightings were recorded in low altitude areas, with 98% (*n* = 13,802) of koalas sighted at altitudes of less than 100 m above sea level (Supplementary Table S6).

Sightings by land category (Fig. 4b and Supplementary Table S6) were highest in residential land (63%), followed by agricultural (15%), and parkland (12%), with sightings on other land types occurring less frequently (educational: 3.5%, commercial: 3.4%, industrial and other land types 3.1%). Koalas in residential areas were predominately observed in the LGAs of Brisbane (59%), Ipswich (14%), Logan (73%), Moreton Bay (70%) and Redland (75%). Koalas on agricultural land, were most frequently observed in the LGA of Ipswich (73%), followed by Brisbane LGA (18%), while other LGAs reported less than 5% of koalas on agriculture land. Between 10 and 14% of koalas were sighted in parklands of the LGAs Brisbane, Logan, Moreton Bay and Redland, while Ipswich LGA reported only 5% of sightings in parklands.

Kernel smoothed density estimates of koala sightings between 1999–2013 in SEQLD are shown in Supplementary Fig. S4. The proportions of the study area with categorized koala sighting densities per square kilometer for 4-year observation periods (based on the kernel density estimation approaches) are summarized in Table 1. Areas where no koalas were sighted decreased over time (from 35% to 21%), while areas with up to one koala sighted increased over the same period (from 64% to 76%). The maximum estimated sighting density was 13.5 koalas per square km.

**Table 1:** Percentage of study area with estimated density of koala sightings per four-year observation period for 1999–2013 in SEQLD, Australia.

Year	1997–2000	2001–2004	2005–2008	2009–2013
Sighting density^a^	Percentage (number of 1 km^2^ cells) of study area			
0	35.0 (20,223)	31.5 (18,200)	26.8 (15,485)	21.5 (12,423)
>0–1	64.0 (36,979)	67.0 (38,713)	71.8 (41,486)	76.5 (44,202)
>1–2	0.4 (231)	0.6 (347)	0.7 (405)	0.8 (462)
>2–3	0.2 (116)	0.3 (173)	0.35 (202)	0.4 (231)
>3	0.4 (231)	0.6 (347)	0.35 (202)	0.8 (462)
Density range	0–9.6	0–7.7	0–5.0	0–13.5
Total area (number of 1 km^2^ cells)	57,780	57,780	57,780	57,780

Koala sighting density was estimated using a Gaussian kernel with a fixed bandwidth. The numbers in parentheses show the number of 1 km^2^ cells corresponding to the percentage.

^a^Estimated number of koalas per square kilometer.

### Spatial bias in koala sightings

To explore the potential spatial bias associated with sightings of koalas at fine spatial scales, koala sighting locations in a section of the Redlands LGA were overlaid with the road network. Koalas were observed closer to roads, suggesting either higher koala density near roads and/or a higher level of search effort by people along roads (Supplementary Fig. S1).

**Figure 5:**
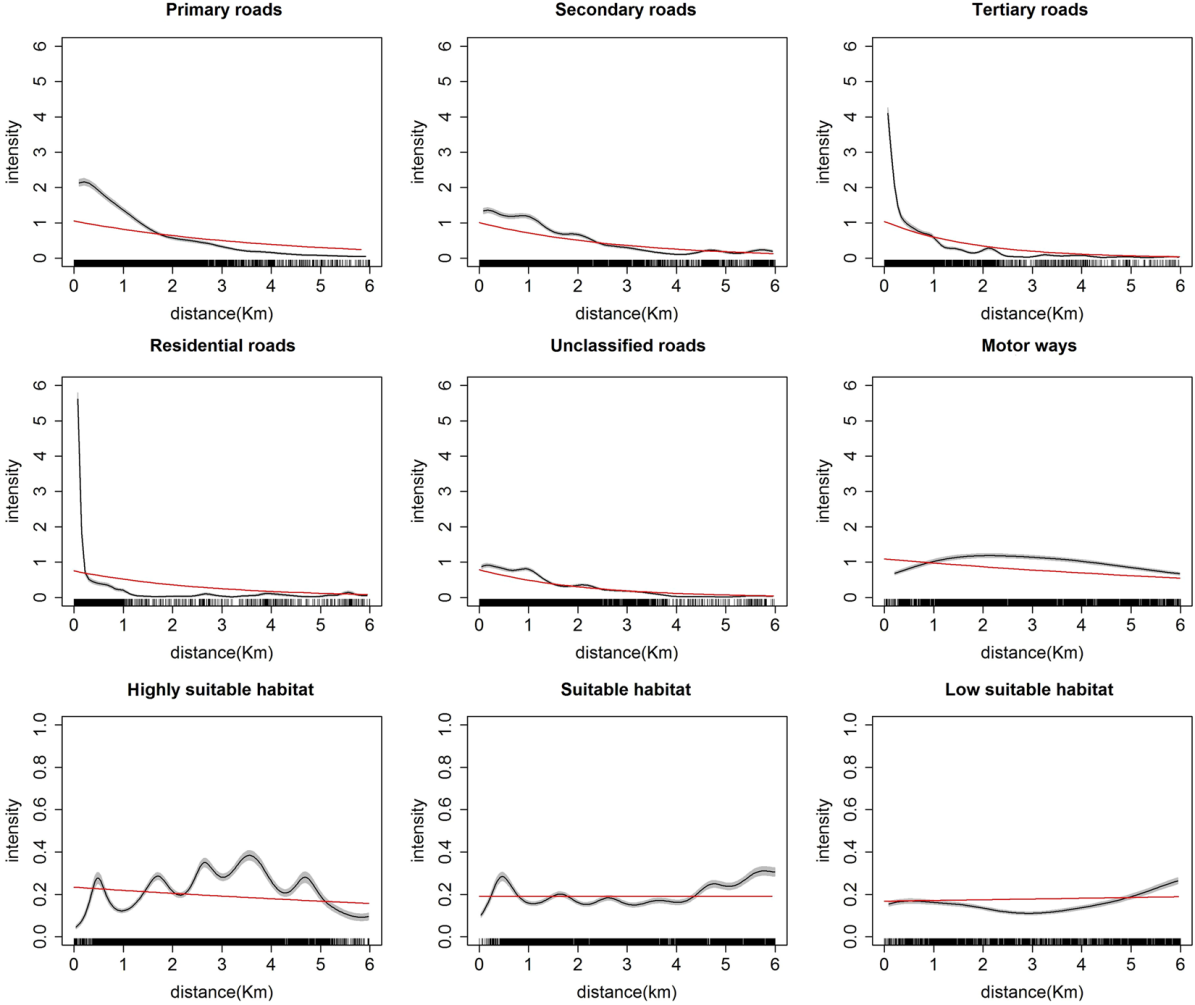
Density of koala sightings (black line with 95% confidence bands -grey) as a function of distance to the landscape features. Estimates of a parametric model using each distance covariate are superimposed on each plot as red lines. The top two panels show koala sighting density as a function of the distance to primary, secondary, tertiary, residential, unclassified roads and motorways. The bottom panel shows koala sighting density as a function of distance to highly suitable, suitable and low suitable habitat. Intensity estimates were calculated using the rhohat function implemented in the contributed R-3.5.0 software (https://cran.r-project.org) package spatstat^34^. Road data were obtained from the Queensland Spatial Catalogue (http://qldspatial.information.qld.gov.au).

To quantify the association between koala sighting density and distance to roads and distance to koala habitat we used the non-parametric rhohat procedure described by Baddeley^34^. We identified a decrease in the density of koala sighting reports with increases in the Euclidean distance from primary, secondary and tertiary roads, with the strongest association observed for tertiary roads (Fig. 5). A similar pattern was also observed for residential roads, but for primary and secondary road types, the density of sightings decreased only gradually as distance from the road increased. Koala sighting density near unclassified roads and motorways was low.

The relationship between sighting density and distance to habitat of different suitability for koalas was not as prominent as it was for distance to roads, thus varying distance to suitable habitat had less of an impact on koala sighting density.

Using the Berman test, we were able to confirm the dependency of sighting density on distance to roads. The magnitude of dependency between sighting density and distance to roads was the highest for tertiary (AUC = 0.87) followed by primary (AUC = 0.85) and secondary roads (AUC = 0.85) (Supplementary Fig. S5 and Table S9). Sighting density was not associated with distance to habitat types of varying suitability for koalas (AUC = 0.43 to 0.49).

By using parametric models, we quantified the mean change in koala sighting density with an increase in distance from roads for each type. For instance, for every one kilometer increase in the distance from a primary, secondary and tertiary road, koala sighting density decreased by a factor of 77%, 71% and 57%, respectively (Supplementary Table S10). Our parametric models identified no statistically significant association between sighting density and the distance to highly suitable, suitable or low suitable koala habitat.

## Discussion

This research is the largest spatial study of koala sightings locations reported by members of the public, representing 17 years of sightings data in an area of 57,780 km^2^. We were able to provide detailed insights into spatial and temporal patterns of koala sightings and estimated koala sighting density, highlighting biases in the sightings of koalas and factors associated with these biases.

Overall, we were able to show that similar spatial patterns and seasonal variations were observed from incidental koala sightings as estimated through systematic surveys of koala populations.

Our analysis indicated seasonal patterns, with a higher number of sightings during the SEQLD koala breeding season, coinciding with an increased koala dispersal and movement of animals seeking mating partners as reported previously^35^. Koala sighting density was low in the Brisbane city area where koala habitat is limited. Koala sighting density was also low in the western inland part of SEQLD, but these estimates are difficult to compare to published koala population data, as only limited active surveys were conducted in these areas and therefore estimates of koala densities have been derived only through extrapolation from statistical models^18^.

Our findings highlight an advantage of involving members of the public in the reporting of wildlife: the incidental koala sightings reported during the study period covered a very large geographical area which would be impossible to cover by systematic surveys alone. Also, interest in reporting was strong. Within each year, on average, a total of 552 individuals reported koala sightings.

A higher number of sightings were reported in residential areas, in particular in low elevation areas close to the coast. These residential areas often contain patchy koala habitat with suitable tree species. When koala habitat is fragmented, koalas have to spend more time on the ground to move from tree to tree searching for food and, in some instances, for resting^31^ therefore the chances of sighting koalas by residents might be higher in this fragmented habitat. Some residential areas are adjacent to dense koala habitat, providing further opportunities for people to see koalas or for koalas to cross into residential areas encompassed by their home range.

Interestingly, in the LGAs with greater numbers of sightings, significant clusters of koala deaths due to vehicle collisions were identified^7^, further highlighting the consequences of spatial intermingling of koalas and humans. The phenomenon of sightings of koalas in urban settings may be correlated with rapid urban development, exposing koalas to areas with high human activity as well as associated threats such as cars and dogs.

Sightings by land type over time needs to be interpreted carefully as land type classifications might have changed throughout the study period. The decrease in the proportion of koala sightings in residential areas compared to other land types from about 80% to 52%, could indicate a reduction in koala habitat in these residential areas over time reducing the koala population. On the other hand, increased sightings from agricultural lands could be attributed to driving of koalas to those areas due to further clearing and fragmentation of habitat^36^. The gradual increase of sightings on parklands may be a result of improved accessibility to parks, more interest by people to report sightings, easier reporting due to technological innovations, or an increase of number of koalas in parklands.

We were also able to identify limitations and inconsistencies in the sightings data of koalas. Although systematic field surveys have shown that the koala population in SEQLD has been rapidly declining in the last 20 years^18^, reports of sightings of koalas by members of the public did not mirror this trend. Instead, sighting numbers increased during this period. It is highly unlikely that increased sightings in the last decade were due to an increase in the koala population or due to an influx of koalas from other areas. Our time series analysis highlighted an increasing trend in koala sightings towards the end of the study period. This could be influenced by the fact that the number of people reporting sightings and the number of koalas reported per person increased over the study period, while the reporting areas expanded. The increase of reporting in some areas may have been influenced by koala conservation initiatives or media reports on koala mortalities that resulted in an increased awareness and more enthusiastic reporting of sightings by some members of the public. Further, the ubiquity of smart phone usage over the last decade may have improved the ease of reporting sightings and could have bolstered sighting numbers. Nevertheless, the increasing reports of sightings indicates a strong public interest in koalas that should be encouraged to support koala conservation initiatives, and strengthens the argument for citizen science to be deployed in wildlife conservation initiatives.

The sudden decrease in koala sightings in 2011 was due to low reporting from Redland and Moreton Bay LGAs, in particular in areas up to 10 km from the coast. Previous research^7^ showed no decrease in koala mortalities reported by members of the public in 2011. Interestingly, though the number of people reporting sightings decreased in Redland and Moreton Bay in 2011, the number of koalas reported per person remained the same, indicating that the people who did not report sightings in 2011 had reported a high number of sightings in the past. The underlining reasons for this variation cannot be explained from the data available but could be related to lower reporting of sightings due to a flood situation prevailing in 2011. It seems unlikely that there would be reduced interest in reporting in this year, however, the building of a railway line in this area resulted in the clearing of koala habitat^37^ and might have resulted in an antagonistic reaction by members of the public and therefore fewer reporting of sightings. On the other hand, perhaps not all reported sightings were entered in the database. This variance highlights a limitation of citizen science data, which can be confounded by irregular reporting or perhaps reporting into different databases, further emphasizing the need to set-up a centralized structured reporting platform for all sightings^33^.

Another limitation of incidental sighting reporting is, that only location information on the presence of wildlife is reported^38^ while information on the absence of wildlife is generally not documented. Due to movements within their home range, wildlife species are not present in the same locations all the time. This is particularly important for wildlife species with large home ranges. Therefore, both presence and absence data as generated through active surveys are usually used for estimating animal populations^18^. Collecting absence records and conducting repeated observations on the same area are useful approaches to improve the quality of sightings data and such measures have partially been incorporated into some citizen science survey programs^39^.

Our analysis showed that koala sightings were spatially biased towards roads. Previous research has also shown that distance to roads is a key factor influencing sightings^39^. Distance to roads is an indication of human activity, represented by human traffic and the presence of housing. We explored different road types and found koala sightings and sighting density was at maximum near tertiary roads. Tertiary roads represent connections between smaller towns and local centers in larger towns. However, the distribution of road types in the study area is not homogeneous, for example motor ways are more extensive in the eastern part of the study area. Interestingly, distance to koala habitat of different suitability was not associated with koala sightings and density. Thus, distance to koala habitat might not be a serious bias in koala sightings. However, it needs to be considered that koala habitat was more scattered over the whole study area, while roads of different types represented line features and were only present in certain parts of the study area.

In areas where accessibility is difficult and/or in low koala density areas, a greater effort has to be made by members of the public to ‘spot a koala’ Thus, a longer time has to be spent to search for a koala and longer distances need to be travelled compared to high koala density and/or easily accessibility areas. In contrast, low numbers of sightings may be reported in certain areas where koala density is high, but accessibility is limited (e.g. protected koala habitat). Hence, this highlights the need to record the actual search effort made by the member of the public to identify a koala.

Finally, in our initial exploration of the dataset we identified several errors in the data, which is a common problem with citizen science data. We identified positional errors (e.g. koala locations in water bodies) or koala locations that did not match the administrative areas specified for corresponding location. Most of these positional errors could be corrected. For example, when street information on the sighting was available, we were able to re-identify the correct location. Positional errors of few meters have only a minimal effect in a distance-based analyses that considers koala home ranges of up to several hectares (depending on the habitat type). However, if high resolutions are used in analyses, positional errors are important and a careful examination of the data to eliminate these errors and appropriate correcting techniques are required^40–42^.

So overall, is citizen science data useful to complement koala population monitoring programs? We would argue that sightings data improves wildlife density estimates generated using systematic surveys. Due to the costs associated with systematic surveys, they are usually limited to small geographical areas and are conducted infrequently. In addition, systematic surveys of koalas focus on pre-identified areas covering specific koala habitat, while areas that are close to residential areas and roads are not included in such surveys, thus questioning if population estimates using survey data alone might also be biased. Thus, the development of approaches to combine systematic surveys with incidental sightings data would be desirable, as sightings can confirm species presence in areas where systematic surveys are not conducted or feasible. In particular, statistically robust analytical methods or the use of external data need to be considered to increase the inferential validity of model-based population estimates derived from incidentally or opportunistically collected wildlife data^27^. Some recent research has described methodologies for combing sightings data with systematic survey data to estimate the density of wildlife species^26,43^. In addition, suitable statistical approaches need to be applied to the analysis of sightings data, considering and estimating search effort and implementing bias correction approaches.

This dataset was not considered appropriate to directly estimate koala populations due to of lack of key search effort information and the lack of species absence records. We recommend collecting data on the search effort of people reporting sightings (e.g. time spent searching for a koala, distance traveled) and conducting observations repeatedly in the same area. In particular, the reporting of absences should be encouraged. This would involve searching for a koala actively and re-visiting absence locations every season to make sure absences are repeated or to report a new sighting in this former absence location. To improve the spatial bias identified, more members of public should be encouraged to join the reporting program so that larger areas can be covered, in particular in less accessible areas and inland where generally fewer sightings are reported. We also recommend that an active koala counting program should be developed in partnership with the public and that it should be conducted at least once a year. Online resources should be provided to members of the public to get acquainted with identification of koalas, koala habitats, and the reporting tools.

Reporting koala location and search effort information is not easily conducted without modern communication tools such as a mobile phone application that could track locations along an observers walking track in defined intervals (for example recording either a sighting or an absence of koala every 5 minutes of travel or every 200 meters along a track). Such applications have been successfully employed in other citizen science programs such as the eBird project. This program collects information regarding the duration and distance travelled while birding, and on the number of people in each birding party^44^. Also, in the South Australian Great Koala Count in 2014^19^ observers were asked to report koalas using a smartphone application, while recording their experience and search effort^39^.

In addition to a reliable reporting tool, an appropriate data storage system to improve the generation and use of koala sightings and citizen data is required. A web-based reporting system can provide members of the public with the option to upload the data captured by the mobile application or to enter the data directly into a web interface. Incidental koala sightings used in this analysis were retrieved from a clinical database (KoalaBASE) and the entry of sightings into this database was terminated in mid-2014. Koala sightings are now being reported to national and local databases and consolidation of this data in a single platform would be highly desirable.

The research presented here is the most detailed analysis of koala sightings to date, with specific attention to the quality and biases of the data collected. Estimation of koala population using this data set requires complex statistical modelling techniques. However, the sightings data presented here can serve as an indication of changes in the koala population and could compliment active koala population monitoring programs.

## Methods

### Study area

**Figure 6:**
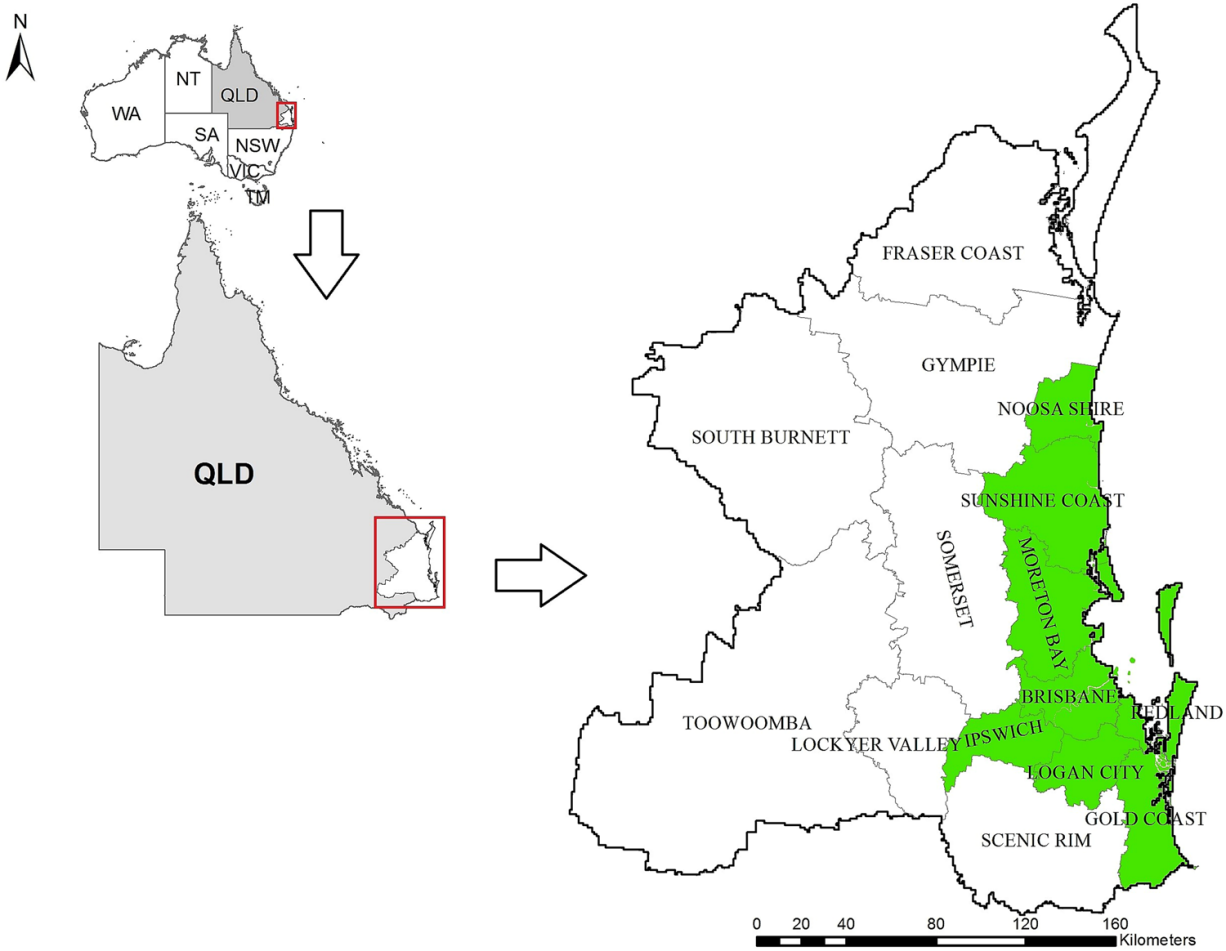
Study area in SEQLD with local government area (LGA) boundaries. LGAs with strong focus on koala conservation are shown in green.

Our study area (Fig. 6) focused on SEQLD and included 15 Local Government Areas (LGAs) and 1,517 suburbs. The total extent of the study area was 57,780 square kilometers which included regions with prominent koala habitat, namely Pine River Shires and the Koala Coast. The eastern parts of the Pine River Shire and the Koala Coast are heavily urbanized with relatively high human population densities. Other parts of the study area towards north and west, and also the metropolitan area of Brisbane city in the central eastern region, have a low koala population density. Approximately 28% of the study area was less than 100 m above sea level, which included most areas up to 20 km from the coast; 15% of the study area was between 100–200 m and 56% were greater than 200 m above sea level (with the latter gradually increasing westwards to a maximum of 1,280 m).

### Koala sighting data

For this study we used geo-referenced records of sightings of koalas between 1997 and 2013 that were stored in KoalaBASE. Our definition for a koala sighting was a live koala sighted by a member of the public, referred to in this paper as an ‘incidental’ koala observation. Records of koalas that were presented to wildlife hospitals with injuries, koalas that were reported dead, or needed to be euthanized were not used in the analyses reported in this paper.

If incorrect or no sighting coordinates (longitude/ latitude) had been stored in KoalaBASE geo-referencing of koala sighting locations was conducted based on the street address where the koala was sighted and longitude and latitude coordinates were determined using Google^®^ maps. Sightings of more than one animal, or joeys of koalas were recorded as separate sighting incidents at the same location. We excluded sightings that were reported more than once in the same location by multiple observers, i.e. representing repeated sightings of the most likely same animal on the same day, and sightings with missing or incorrect coordinates. We also retrieved from KoalaBASE the sex and size or age of the sighted koala (such as adult, sub adult and young) and details on the physical location where the koala was spotted.

### Spatial data

The spatial information listed below was retrieved from the Queensland Spatial Catalogue (http://qldspatial.information.qld.gov.au).

### Land types

We hypothesized that the number of koala sightings depended on the land type where koalas were located. Land type data were obtained at the mesh block level, which is the smallest geographic region in the Statistical Geography Standard in Australia. Land type represents the ‘usage’ of land and is classified as residential, agricultural, industrial, commercial and educational land or as parkland. Different land types were processed as separate digital map files and joined with koala sightings locations to calculate number of koala sightings in different land types for different study time periods.

### Elevation

We considered that elevation of the land influenced vegetation and thereby koala habitat. Elevation was classified into altitudes of <100 m, >100–200 m and >200 m and the number of koalas sighted in each elevation class was calculated.

### Distance to road types

We hypothesized that different road types provided varying access to koala habitat and therefore represented a key factor influencing the frequency of koala sightings. The road network was classified as primary, secondary, tertiary, residential, motorways and unclassified roads. For each road type, the Euclidian distance to the nearest road was calculated using the distmap function in the contributed spatstat^34^ package in R. Raster maps were prepared for each road type representing the minimum distance to nearest roads (Supplementary Fig. S2a).

### Distance to koala habitat of different suitability

We further hypothesized that the distance to koala habitat and type of koala habitat were associated with the reporting of koala sightings by members of the public. For example, koalas would be more frequently sighted in close proximity to habitat suitable for koalas. Broad vegetation groups (BVGs) which represent a higher-level grouping of vegetation communities in Queensland, based on water salinity, landscape and dominant tree species, can be used do classify habitat types^45^. We obtained spatial data on 98 BVG’s at a regional level of 1:1,000,000 and used the method proposed in a recent study^18^ to classify the suitability of koala habitat into three groups (highly suitable, suitable, low suitable), based on tree types present in the BVGs. The same method as for distance to roads above was used, to calculate the Euclidian distance to highly suitable, suitable and low suitable habitat and raster maps were prepared (Supplementary Fig. S2b distance to habitat suitability categories). The distance to roads and habitat suitability raster maps were prepared in 1 km^2^ resolution and cropped to match the extent of the study area.

### Data analysis

R software^46^ version 3.5.0 and Microsoft Excel® 2016 was used for data analysis and ArcMap^47^ version 10.5 for mapping.

First, temporal patterns of sighting events were quantified by calculating sightings frequencies per year and weekly and monthly frequencies aggregated to four time periods (1997–2000, 2001–2004, 2005–2008, and 2009–2013). We also determined how many individuals had reported a koala sighting each year. We then calculated the frequency of sightings by land types, LGAs and distance to the coast for the four time periods. We conducted a time series analysis using the entire koala sightings dataset using the stats package in R^46^.

Secondly, spatial and spatial-temporal patterns of sightings were summarized; including the spatial distribution of sightings in varying distance (0–20 km with 5 km increment using the Buffer tool in ArcMap) from the east coast of Queensland and in varying altitudes for the four time periods.

Thirdly, we estimated koala-sighting density using Gaussian kernel estimation using a fixed bandwidth. Kernel density estimation represents a non-parametric method to estimate the density of point data^48^. We used Scott’s rule of thumb method to calculate the bandwidth for the kernel density estimations^34^. We then calculated the proportion of the 57,780 one-square kilometer grid cells covering the study area that had a specified koala density per km^2^. For this we used the kernel density raster image and reclassified it into categories of koala density per km^2^ ranging from 0 to more than 3 koalas per km^2^ (using the Reclassify and Zonal geometry tools in ArcMap). The spatstat^34^ package in R was used for kernel density estimations and all other statistical analysis as described hereinafter.

We explored graphically the relationship between koala sighting density and each of the distance covariates using a non-parametric method. For this, we treated sightings locations as a realization of a spatial point process in the study area. The intensity^34^ (*λ*) of a spatial point process at location (*u*) depends on a spatial covariate *Z*(*u*) and is characterized by the relationship *λ*(*u*) = *ρ*(*Z*(*u*)). Rho is proportional to the ratio of density of covariate value at the points of the observed point process to the density of covariate value at random locations of the study area. We used the rhohat function in spatstat technique to quantify the association between koala sighting intensity and each spatial covariate as described by Baddeley, *et al*.^34^, assuming that koala sightings follow an inhomogeneous Poisson point pattern, where points are independent with same the probability distribution in any small area of space. The rhohat function in spatstat uses a kernel smoothing method to estimate intensity across space.

We then evaluated the statistical significance of the dependency between koala density and the spatial distance covariates using the Berman test^49^. The null hypothesis for the Berman test is that point locations of sightings do not depend on the distance to roads (or habitat), while the alternative hypothesis states that locations of sightings depend on the distance to roads (or habitat). The Berman test evaluates the observed distribution of covariate values at sighting locations over distribution of covariate values at all locations.

We summarized the magnitude of this dependency between densities of koala sightings and spatial distance covariates. For this, we constructed receiver operator characteristic (ROC) curves and calculated the corresponding areas under the curve (AUC) for the spatial point process, which describes, for a given fraction of study area, the observed percentage of sightings. This is similar to drawing a varying distance of buffers on either side of the line of the distance to roads covariate (raster) and calculating the percentage of sightings falling within it. An AUC of 1 indicates a strong discriminant ability whereas 0.5 indicates lack of discriminatory power of the covariate on sightings density.

Finally, we quantified how koala sighting density changed with each unit change in the distance covariates. For this analysis we used parametric models assuming a log linear relationship between density of koala sightings and each distance covariate^34^. The models were developed using ppm function of spatstat package of R. Again, the discriminant ability for each distance covariate was assessed by generating AUC values.

## Supplementary information

Supplementary Information

## Data Availability

The incidental koala sighting dataset analyzed in this study is available from the 10.6084/m9.figshare.7603481.
