# Supplementary material for: The value of long-term citizen science data for monitoring koala populations
Source: Sci Rep. 2019 Jul 11;9:10037. doi: 10.1038/s41598-019-46376-5 (PMC6624211; doi:10.1038/s41598-019-46376-5)
Supplement: Supplementary file 1 — Supplementary Information [file 41598_2019_46376_MOESM1_ESM.pdf]

Supplementary information

The value of long-term citizen science data for monitoring koala populations

Authors

Ravi Bandara Dissanayake[1 *], Mark Stevenson[2], Rachel Allavena[1] & Joerg Henning[1]

[1] School of Veterinary Science, University of Queensland, Gatton Queensland 4343, Australia.

[2] Faculty of Veterinary and Agricultural Sciences, University of Melbourne, Parkville Victoria 3010, Australia.

* Ravi Bandara Dissanayake: r.dissanayake@uq.edu.au

Figures

S1. A section of Redland LGA showing road network (black lines) and locations of sighted koalas (red dots), between 2002-2004. Figure was produced using ESRI ArcGIS 10.5 (http://www.esri.com/software/arcgis).

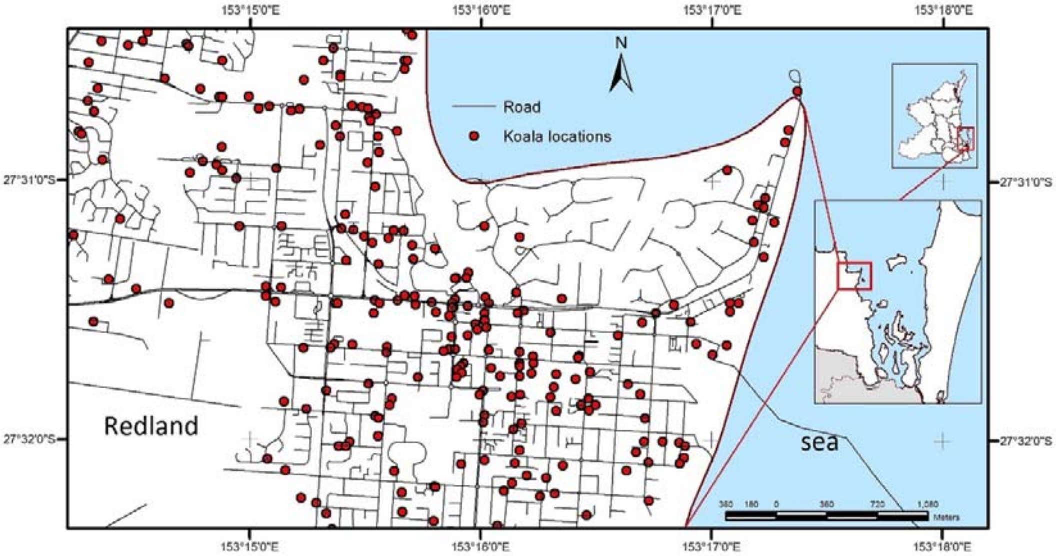

S2. Raster maps showing distance to nearest road types (a) and distance to nearest habitat suitability types (b) in the study area in SEQLD, Australia. The legend shows the distance in metres. Figure was produced using ESRI ArcGIS 10.5 (http://www.esri.com/software/arcgis).

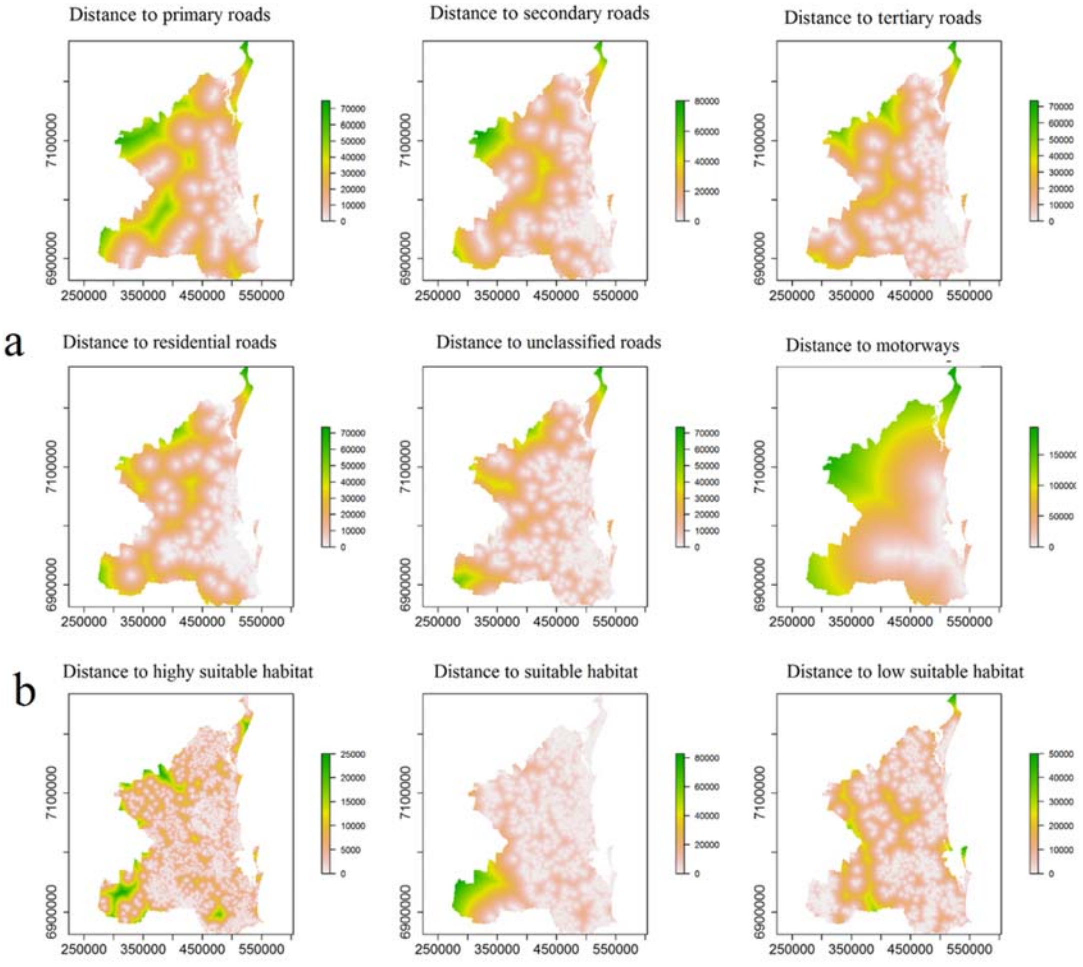

S3. Koala sightings reported per week in SEQLD, Australia by time period between1997-2013.

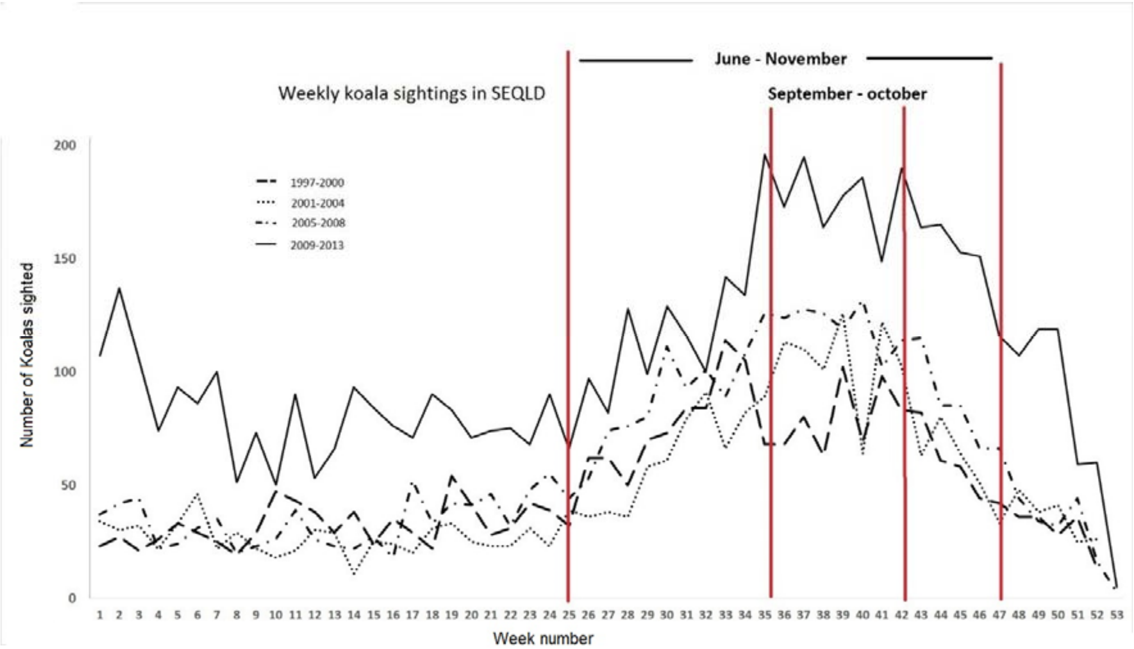

S4. Kernel smoothed density of koala sightings by time year period, from 1997-2013 in SEQLD, Australia. The legend show the number of koalas sighted. (Table 1 for more details). Figure was produced using R software(R Core Team, 2017) version 3.5.0. (Table 1 for more details).

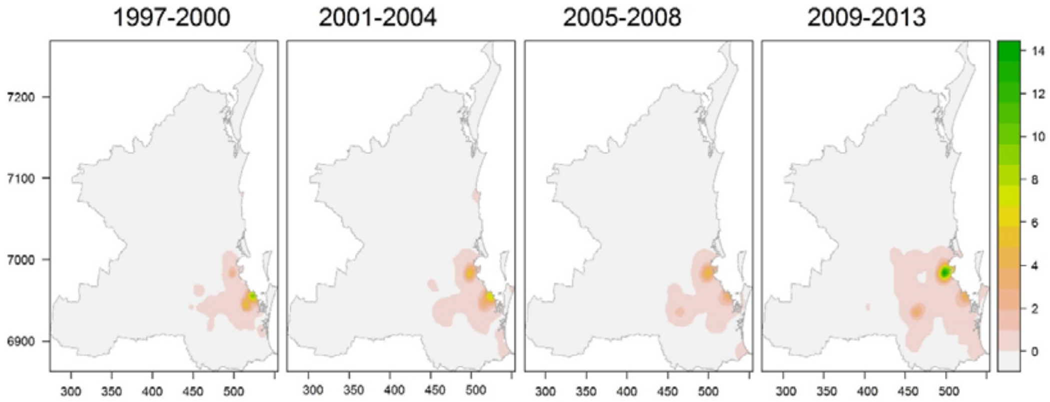

S5. The ROC curves for koala sighting density against distance to nearest feature (roads or habitat suitability type). Horizontal axis shows proportion land area (ρ) and vertical axis shows proportion of sightings (roc(ρ)). Figure produced using spatstat package in R software (R Core Team, 2017) version 3.5.0.

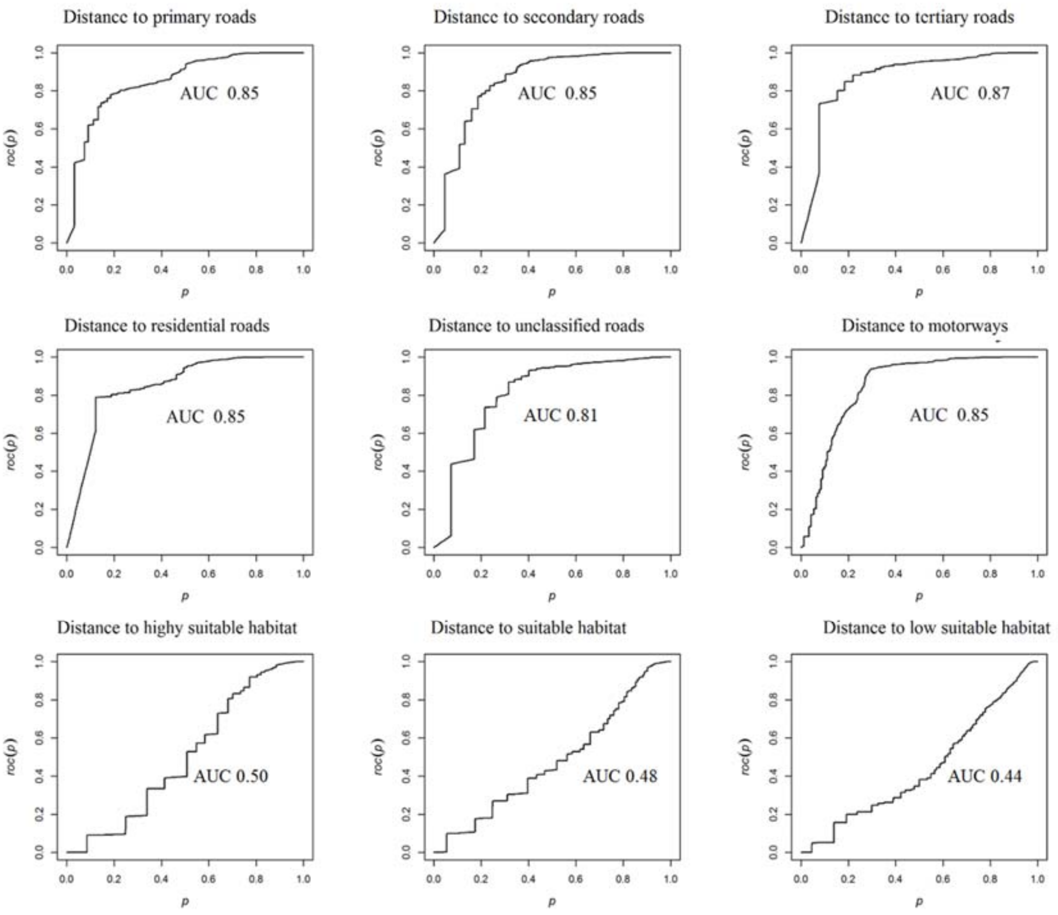

Tables

S6. Counts and percentages of koala sightings in different distances to coast and in different land types by time periods, from 1997-2013 in SEQLD, Australia. Percentages are in parenthesis (Total number of sightings N= 14076).

**Feature**		**1997-2000**	**2001-2004**	**2005-2008**	**2009-2013**
Distance to coast (Km)	0-5	951 (37.50)	1,086(33.75)	731(28.22)	1,322(23.06)
	5-10	577 (22.75)	1,040(32.32)	903(34.86)	1,943(33.90)
	10-15	541 (21.33)	593(18.43)	388(14.98)	899(15.68)
	15-20	248 (9.78)	230(7.15)	76(2.93)	144(2.51)
	>20	219 (8.64)	269(8.36)	492(19)	1,424(24.84)
Land type	Residential	1,948 (76.81)	2,359(73.31)	1,591(61.43)	2955(51.55)
	Agricultural	136 (5.36)	281(8.73)	449(17.34)	1,226(21.39)
	Parkland	229 (9.03)	306(9.51)	287(11.08)	890(15.53)
	Commercial	91 (3.59)	109(3.39)	105(4.05)	181(3.16)
	Other	132 (5.21)	163(5.07)	158(6.10)	48(08.37)

S7. Counts and percentages of koala sightings reported in each local government areas (LGA) by time periods between 1997-2013 in SEQLD, Australia. Percentages are in parenthesis (Total number of sightings N= 14076).

	**LGA name**	**1997-2000**	**2001-2004**	**2005-2008**	**2009-2013**
Coastal LGA from north to south	Noosa	1 (0.04)	7 (0.22)	0 (0.00)	0 (0.00)
	Sunshine Coast	0 (0.00)	2 (0.06)	1 (0.04)	4(0.07)
	Moreton Bay	403 (15.89)	1,201(37.32)	1,295 (50.00)	3,264 (56.94)
	Brisbane	183 (7.22)	248 (7.71)	141 (5.44)	270 (4.71)
	Redland	1,127 (44.44)	1,064 (33.06)	556 (21.47)	642 (11.20)
	Gold Coast	6 (0.24)	28 (0.87)	23 (0.89)	67 (1.17)
Inland LGA north to south	Somerset	11 (0.43)	8 (0.25)	14 (0.54)	165 (2.88)
	Ipswich	101 (3.98)	170 (5.28)	401 (15.48)	968 (16.89)
	Logan	695 (27.41)	475 (14.76)	140 (5.41)	207 (3.61)
	Lockyer Valley	1 (0.04)	0 (0.00)	2 (0.08)	17 (0.30)
	Scenic Rim	6 (0.24)	7 (0.22)	5 (0.19)	98 (1.71)
	Toowoomba	2 (0.08)	8 (0.25)	12 (0.46)	30 (0.52)

S8**.** Number of koala sightings reported in each LGA from 1997 to 2013 in SEQLD Australia.

**Row Labels**	**1997**	**1998**	**1999**	**2000**	**2001**	**2002**	**2003**	**2004**	**2005**	**2006**	**2007**	**2008**	**2009**	**2010**	**2011**	**2012**	**2013**	**Grand Total**
Noosa				1	1	6											1	9
Sunshine Coast						2			1				2	1	1			7
Moreton Bay	51	109	48	195	156	353	391	301	323	277	306	389	791	1019	125	1001	327	6162
Brisbane	40	43	27	73	49	55	58	86	50	23	39	29	62	83	15	74	36	842
Redland	233	210	268	416	260	358	185	261	137	104	113	202	262	239	8	90	43	3389
Gold Coast			1	5	3	8	7	10	4	3	1	15	26	18	5	7	11	124
Somerset	3	2	2	4	1	5	1	1		2	5	7	14	7	28	97	19	198
Ipswich	37	33	13	18	7	11	30	122	136	77	104	84	315	263	148	144	98	1640
Logan	161	171	123	240	159	163	70	83	36	28	25	51	47	53	3	72	32	1517
Lockyer Valley			1						1	1			2	2	3	6	4	20
Scenic Rim	2	2		2		2	4	1	2		2	1	21	11	17	30	19	116
Toowoomba				2		4	2	2	1		9	2	19	4		6	1	52
**Grand Total**	**527**	**570**	**483**	**956**	**636**	**967**	**748**	**867**	**691**	**515**	**604**	**780**	**1561**	**1700**	**353**	**1527**	**591**	**14076**

S9**.** Dependency of koala sightings on each covariates was assessed by Berman test, and magnitude of the dependency was assessed by constructing ROC curve and calculating area under the curve (AUC). The highest discriminant ability is observed in distance to nearest tertiary road.

**Spatial covariate**	**Berman test P value**	**Area under the ROC curve (AUC)**
distance to primary roads	0.00	0.85
distance to secondary roads	0.00	0.85
distance to tertiary roads	0.00	0.87
distance to residential roads	0.00	0.85
distance to unclassified roads	0.00	0.81
distance to motorways	0.00	0.85
distance to highly suitable habitat	0.00	0.50
distance to suitable habitat	0.90	0.48
distance to low suitable habitat	0.00	0.44

S10**.** Results of parametric models. Koala sighting density was fit as a log linear function of each distance covariates separately (Effects of each model is shown in fig. 6 as red lines). The highest discriminant ability is observed in distance to nearest tertiary road.

Model	Coefficient (log scale)	CI 95.lo	CI 95.hi	% change in density for every km increase in the predictor	Ztest	AUC
distance to primary roads	-0.25	-0.26	-0.24	0.77	***	0.86
distance to secondary roads	-0.34	-0.35	-0.33	0.71	***	0.88
distance to tertiary roads	-0.55	-0.57	-0.54	0.57	***	0.90
distance to residential roads	-0.37	-0.38	-0.35	0.69	***	0.90
distance to unclassified roads	-0.48	-0.50	-0.46	0.62	***	0.84
distance to motorways	-0.11	-0.12	-0.11	0.89	***	0.85
